# Malnutrition: laboratory markers vs nutritional assessment

**DOI:** 10.1093/gastro/gow013

**Published:** 2016-05-11

**Authors:** Shishira Bharadwaj, Shaiva Ginoya, Parul Tandon, Tushar D. Gohel, John Guirguis, Hiren Vallabh, Andrea Jevenn, Ibrahim Hanouneh

**Affiliations:** ^1^Department of Gastroenterology/Hepatology, McMaster University, Hamilton, Ontario, Canada; ^2^Department of Gastroenterology/Hepatology, Cleveland Clinic, Cleveland, OH, USA

**Keywords:** malnutrition, physical examination, serum markers

## Abstract

Malnutrition is an independent risk factor for patient morbidity and mortality and is associated with increased healthcare-related costs. However, a major dilemma exists due to lack of a unified definition for the term. Furthermore, there are no standard methods for screening and diagnosing patients with malnutrition, leading to confusion and varying practices among physicians across the world. The role of inflammation as a risk factor for malnutrition has also been recently recognized. Historically, serum proteins such as albumin and prealbumin (PAB) have been widely used by physicians to determine patient nutritional status. However, recent focus has been on an appropriate nutrition-focused physical examination (NFPE) for diagnosing malnutrition. The current consensus is that laboratory markers are not reliable by themselves but could be used as a complement to a thorough physical examination. Future studies are needed to identify serum biomarkers in order to diagnose malnutrition unaffected by inflammatory states and have the advantage of being noninvasive and relatively cost-effective. However, a thorough NFPE has an unprecedented role in diagnosing malnutrition.

## Introduction

Malnutrition presents a substantial socioeconomic challenge in today’s healthcare landscape with an estimated prevalence of 30–50% [1–7]. The prevalence may be even higher in long-term care facilities, where it has been reported to be as high as 85% [1–7]. However, due to historical inconsistencies in defining and identifying malnutrition, the actual prevalence in the population is unknown [8]. Furthermore, malnutrition has been associated with increased healthcare-associated costs including longer hospital length of stay (LOS) and increased rates of major and minor complications [9]. Looking at just eight diseases (chronic obstructive pulmonary disease [COPD], coronary artery disease, breast cancer, colorectal cancer, depression, dementia, stroke, and musculoskeletal disorders), the economic burden of their associated malnutrition has been estimated to be around US $157 billion, with COPD having the highest economic burden out of those [10]. Additionally, malnourished patients have a longer LOS at healthcare facilities by an average of 11 days. Moreover, they were found to have higher rates of readmission or require ongoing services such as home healthcare following discharge [11]. Hence, due to the recognition of the importance of the impact of malnutrition on economic burden, the Centers for Medicare and Medicaid Services (CMS) have implemented a change in their reimbursement rates according to its severity [5].

Malnutrition is associated with an increased risk of major and minor complications as well as an increase in direct and indirect costs. One retrospective study of 709 adult patients from 25 Brazilian hospitals reported that the incidence of complications in the malnourished was 27% (relative risk [RR] = 1.60) compared with17% in the well-nourished counterparts [12]. Furthermore, mortality in the malnourished patients was 12.4% *vs* 4.7% in the well-nourished patients (RR = 2.63). Similarly, another study of 104 patients with acute stroke of < 24 hours duration reported that malnourished patients were more likely to have higher stress reaction, increased frequency of infections and pressure ulcers compared with the appropriately nourished group [13]. Additionally, disease-associated malnutrition results in an increased number of missed days from work and therefore lower economic productivity [14]. Indirect costs related to care provided by family members and the subsequent effect on their socioeconomic activities cannot be accurately determined [10]. Nevertheless, the impact of malnutrition on our society is substantial, and accurate diagnostic measures are necessary to identify those at risk.

A major obstacle for diagnosing malnutrition is the lack of a unified definition of the term. Furthermore, there is no standard method for screening and diagnosing patients with malnutrition, leading to confusion and varying practices among physicians across the world. The appreciation that inflammation plays a major role in the pathophysiology of malnutrition is also lacking. Overall, this has led to a lot of misdiagnoses and a general under-recognition of the importance of malnutrition [2]. An all-encompassing definition of malnutrition has been proposed by Jenson *et al.* as “decline in lean body mass with the potential for functional impairment” [1]. This impairment can be at different levels of function ranging from molecular to gross motor. They also realized the role of inflammation leading to either acute or chronic disease-related malnutrition. Non-inflammatory states such as chronic starvation and anorexia nervosa are also recognized as separate categories of malnutrition [1].

There are a number of tools that are used to assess the characteristics of malnutrition. This paper will focus on two of the frequently used malnutrition assessment tools—laboratory data (serum markers) and physical examination (nutritional assessment)—and compare them to assess which is more useful in a clinical setting.

## Laboratory/Serum Markers

**Figure 1.**
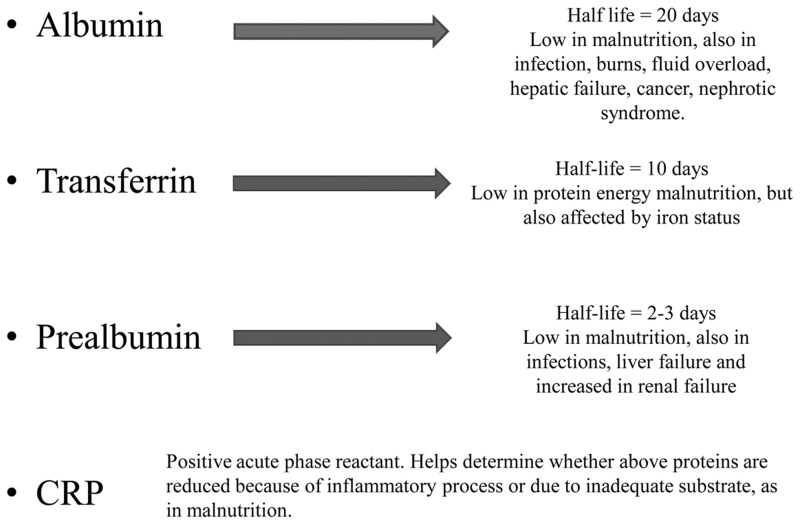
Laboratory markers

Historically, serum proteins such as albumin and prealbumin (i.e. transthyretin) have been widely used by physicians to determine patients’ nutritional status (Figure 1). Other markers that have been studied include retinol-binding protein (RBP), transferrin, total cholesterol and indicators of inflammation such as C-reactive protein (CRP) and total lymphocyte count (TLC). These markers and their role in assessing malnutrition will be discussed in this section.

### Albumin

Albumin is a serum hepatic protein with a half-life of 14–20 days. It functions as a carrier molecule for various minerals, hormones and fatty acids and also helps to maintain oncotic pressure in the capillaries [15]. More than 50% of its total pool is located in the extravascular compartment, and only a minority of it (about 5%) is produced by the liver daily. Thus, a patient’s protein consumption in a day has hardly any effect on the patient’s albumin level [16]. However, albumin is characterized as a negative acute-phase protein, and its pool is affected by a number of inflammatory conditions and drugs, especially those that affect liver function. For example, hepatic failure, burns, sepsis, trauma, post-surgery states and cancer have all been shown to decrease albumin levels.

The concept of “stress-induced hypoalbuminemia” is somewhat controversial, but it demonstrates the concept of the body’s normal physiological response to injury. Since there are a multitude of disease processes that alter the level of albumin, it becomes an unreliable serum marker for malnutrition. This is especially true in acute healthcare settings, where a number of infectious and inflammatory states alter the serum concentration of albumin. Despite this, physicians frequently rely on albumin levels to gauge a patient’s nutritional status.

However, there is some contrary evidence that measuring albumin levels may be a useful tool for diagnosing malnutrition, especially in cardiac transplant and orthopedic patients. In one study of 60 cardiac transplant recipients at least five years post transplant, serum albumin was found to be a better predictor of underlying malnutrition than body mass index (BMI) and subjective global assessment (SGA) [17]. Similarly, serum albumin has also been used during preoperative management to screen and treat at-risk patients undergoing elective orthopedic surgery to reduce postoperative complications [18].

The evidence is weak for using serum albumin as a marker of malnutrition in non-inflammatory states such as starvation. A meta-analysis of 63 studies, which included 2125 patients and investigated the effects of starvation on serum albumin levels in otherwise healthy individuals, reported that the levels remained normal until the patients reached extreme states of starvation (BMI < 12 or length > 6 weeks of starvation) when the malnutrition was already physically evident. Hence, it was concluded that albumin cannot be reliably used as a marker for diagnosing protein-calorie malnutrition [19]. Similarly, a case-control study that compared 14 anorexia nervosa patients with 15 healthy subjects reported that serum albumin levels did not vary in individuals with anorexia nervosa compared with controls during a follow-up of one year [20].

There has been much debate regarding the role of albumin as a measure of nutritional status in the non-acutely ill geriatric population with low functional capacity. One study found that using serum albumin levels < 3.5 g/dL as the sole parameter for malnutrition would have low specificity for identifying nutritional status in the functionally impaired elderly [21]. It would result in up to 80% of the individuals being wrongfully diagnosed as malnourished. Additionally, posture-related effects (supine, sitting, standing and moderate exercise) on serum albumin levels have also been reported [22]. This is related to the alterations in hydrostatic and oncotic pressures with changes in body positions [23]. This can lead to falsely elevated or diminished levels of albumin; due to the above-mentioned reasons, albumin level is not the most accurate measure for determining malnutrition in this patient population.

### Prealbumin, transferrin and retinol-binding protein

Similar to albumin, prealbumin (PAB) is also a negative acute-phase protein produced by the liver. Thus, it is affected by some of the same inflammatory states such as infections and liver disease. However, there are a few key differences between these two proteins. The half-life of PAB is much shorter (2–3 days), and its total body pool is considerably smaller than albumin. Both of these factors theoretically allow it to be used as a more reliable indicator of acute changes in a patient’s nutritional status. However, PAB is degraded by the kidneys, and consequently any renal dysfunction causes an increase in its serum levels. Furthermore, one of the functions of PAB is to act as a transport protein for thyroxine. In hyperthyroid states, the molecules of prealbumin are saturated with thyroxine, and hence the measured serum levels of PAB are low. Similarly, PAB levels are high in hypothyroid states [16].

Transferrin is a serum protein and yet another negative-phase reactant that has been used to determine nutritional well-being [9]. Inaccuracies can result from this method because of transferrin’s role in iron transport. In iron-deficiency states (including chronic blood loss anemia), the levels of transferrin are elevated due to increased amount of iron absorption. Consequently, the levels are decreased in iron-overload states [24]. One study, which compared 44 underweight patients with 69 normal or overweight elderly subjects, reported that there was no correlation between fat-free mass and transferrin levels, making it a poor serum marker for assessing malnutrition [25]. Like PAB, transferrin levels also increase with renal failure. Oral contraceptives or estrogen formulas also alter serum transferrin levels [16].

RBP mainly exists as a part of the retinol-circulating complex. Vitamin A and zinc are vital in the proper functioning of RBP, and hence any abnormalities in the levels of these micronutrients affect the levels of RBP in the serum. Also, the entire complex is degraded by the kidneys, and thus renal insufficiency increases the levels of RBP [26]. One study of 34 obese patients investigated the effects of very low-calorie diets (< 500kcal/d) on serum albumin, PAB and RBP levels over 20 days. While the levels of serum albumin did not change, PAB and RBP were shown to decrease significantly. This demonstrates that PAB and RBP are better tools for evaluating short-term effects of nutritional modifications because of their rapid turnover. On the contrary, albumin has a much larger body pool and longer half-life; therefore, its levels do not respond quickly to the restricted diets [27]. However, another study of 24 postoperative patients reported that short-lived proteins such as PAB and RBP are not useful for nutritional assessment in postsurgical patients [28]. The reason stated was that they are both influenced by the metabolic stress response post surgery. On the contrary, Nataloni *et al.* investigated the role of PAB in 45 consecutive head-injury patients admitted to the intensive care unit (ICU) and found that PAB was the most sensitive serum marker for the early diagnosis of malnutrition and for assessing the appropriateness of the nutritional therapy for malnourished patients [29]. Similarly, Erstad *et al.* reported that PAB was a better indicator than albumin for assessing the adequacy of postoperative nutritional support [30]. They found that PAB levels rose quickly to normal range after the administration of parental nutrition, as compared with albumin, and that it may also be an effective means for determining both nutritional status and response to therapy as well as avoiding unnecessary increase in supplemental caloric intake and excessive laboratory testing.

### Other serum markers

Because of the recent advancements in understanding the role of inflammation in malnutrition, several inflammatory mediators have been studied as serum markers. One such molecule is CRP, which is a positive acute-phase reactant [31]. However, CRP levels can be mildly elevated at baseline in approximately one-third of the American population. Also, non-nutritional factors (e.g, cardiovascular disease) and other inflammatory states (e.g. infections) can affect the levels of CRP [32].

TLC is another popular serum marker with proposed usefulness for determining nutritional status. Levels of TLC have been shown to vary with the degree of malnutrition. Levels < 1500/mm^3^ correlate well with malnutrition, and those < 900/mm^3^ reflect severe malnutrition [33,34]. However, a study of 161 elderly subjects reported that TLC was not a good marker of malnutrition in the elderly population. They reported that TLC was more “reflective of age rather than nutritional status” [35].

### Nitrogen balance

The historical gold standard for assessing protein intake is nitrogen balance. It is calculated as nitrogen intake minus nitrogen loss from the body and is useful for evaluating protein metabolism because nitrogen is an essential part of protein building blocks– amino acids. A negative nitrogen balance means there is more loss than intake, which can be used as a marker for assessing malnutrition [36]. Nitrogen balance can be studied by measuring the concentration of urea in the urine. Another technique is to calculate the urinary creatinine/height index. Values of 60–80% and 40% indicate mild and severe protein malnutrition, respectively. However, the drawback with this technique is that the collection of 24-hour urine creatinine is cumbersome [37].

## Nutritional Assessment

The nutrition-focused physical examination (NFPE) is an essential component for diagnosing malnutrition. Focusing on general characteristics such as edema, muscle wasting and subcutaneous fat loss to specific micronutrient related deficiencies, the NFPE is very sensitive for assessing nutritional status. The SGA is a well-validated tool for assessing malnutrition, especially in hospitalized patients. It includes several physical examination assessments such as muscle wasting and subcutaneous fat loss [4].

### Subjective global assessment

The SGA is a well-validated, bedside tool for recognizing malnutrition [4,38–43].The SGA assesses nutritional status based on features of the history and physical examination and scores patients on a scale ranging from well-nourished to severely malnourished (Table 1). Historically, the SGA was proposed to predict postoperative infectious complications. However, since the 1980s, it has transitioned into the gold-standard tool for complete nutritional assessment in patients undergoing hemodialysis and organ transplantation as well as patients diagnosed with gastrointestinal and gynecological malignancies and chronic kidney disease [44]. The value of the SGA for nutritional assessment is the inclusion of the physical examination in its scoring system. In a prospective study of 154 patients with esophageal cancer, only the physical examination components of SGA (e.g. loss of subcutaneous fat, muscle wasting and edema) were significantly associated with malnutrition. This suggests the major importance of physical examination for assessing and diagnosing malnutrition.

**Table 1.** Subjective global assessment

**History**
Weight loss in last six months
Changes in dietary intake
Gastrointestinal symptoms
Functional capacity
Disease and its relation to nutritional requirements
**Physical examination**
Subcutaneous fat
Muscle wasting
Ankle edema
Sacral edema
Ascites

Several studies have employed the SGA in their patient cohorts and reported great validity and reliability in diagnosing malnutrition [45–48]. Hirsch *et al.* performed and validated the SGA on 175 patients admitted to the medical-surgical gastroenterology service [45]. They also reported a 79% inter-rater reliability between assessments made by residents and nutrition specialists and confirmed the usefulness of SGA even when used by inexperienced professionals. Furthermore, Sacks *et al.* recruited 53 patients from long-term care facilities and reported a significant correlation between SGA scores and nutritional complications including death [46]. Hence, SGA classification was deemed to be a cost-effective, noninvasive tool for monitoring nutritional status in geriatric patients. The sensitivity and specificity of SGA for diagnosing malnutrition in these patients were 82% and 72%, respectively.

Since Detsky *et al.* introduced the original SGA, there have been numerous modifications and versions proposed for a variety of reasons [4]. The patient-generated subjective global assessment (PG-SGA) has been proposed for patients with malignancies, acute ischemic stroke and hemodialysis [49–53]. It includes additional questions regarding the presence of nutritional symptoms and short-term weight loss, which can be completed by the patient with the physical examination being performed by a healthcare professional. A score ≥ 9 indicates the need for nutritional intervention. In 71 cancer patients, Bauer *et al.* reported that the PG-SGA score had a sensitivity and specificity of 98% and 82%, respectively, for predicting SGA classifications [49]. They concluded that the PG-SGA accurately assesses nutritional status in patients hospitalized with cancer-related complications. Furthermore, Desbrow *et al.*, in their study of 60 patients on hemodialysis, reported that a PG-SGA score ≥ 9 had a sensitivity and specificity of 83% and 92, respectively, for predicting SGA classification [51]. There were significant correlations between the PG-SGA score and serum albumin and the PG-SGA score and percentage weight loss over the past 6 months. Hence, the PG-SGA score is thought by some authors to be the most appropriate tool for identifying nutritional derangements in gynecological cancer patients.

Other versions of the SGA include the dialysis malnutrition score and malnutrition-inflammation score (MIS). The MIS is unique in that laboratory measures such as serum albumin and total-iron binding capacity are also included in the standard SGA. Few studies have reported the MIS score having significantly higher correlations with actual nutritional status of patients as compared with the conventional SGA [54,55]. However, further studies are required to determine the reliability and validity of these novel nutritional risk assessment tools.

### Assessment of muscle mass and subcutaneous fat

A decline in subcutaneous fat and overall body muscle mass is a significant indicator of malnutrition [56,57]. As part of the aforementioned SGA nutritional tool, muscle mass and assessment of subcutaneous fat are vital for detecting high-risk patients for early intervention. In a study of 138 Crohn’s disease patients with severe malnutrition, preoperative skeletal muscle percentage > 24.3% was the only significant protective factor against postoperative complications and mortality [58].

The pathophysiology of malnutrition-related muscle atrophy is fairly well-delineated. During times of severe nutritional derangements and stress, decreasing glucose concentrations result in decrease in insulin and increase in glucagon levels [59]. This stimulates breakdown of adipocyte cells and increase in free fatty acids and ketones. Ketones are then used as the primary source of energy. Concurrently, amino acids are released from myocyte breakdown and transported for hepatic gluconeogenesis. Subsequently, acute phase proteins are produced by the liver with resultant decreased muscle mass. Furthermore, inflammatory cytokines also signal for muscle degradation and halt myocyte repair mechanisms. Due to these processes, lean muscle tissue and overall skeletal muscle stores are depleted.

Evaluations of muscle mass and subcutaneous fat tissue have been reported to be reliable, as have noninvasive tests for assessing nutritional status. In a cross-sectional study of 262 patients referred to nutritional support teams, 94% of the patients had previous physical examinations to determine loss of fat and muscle mass, which indicate regular use of nutritional assessment in clinical care settings for the diagnosis of malnutrition prior to the referral [60]. Anthropometric measurements of triceps skinfold thickness and upper arm circumference have been used to accurately estimate body muscle mass [61–66]. The triceps skinfold thickness is measured at the midpoint between the acromion process and olecranon process, and measured values are compared with standardized values previously determined. Importantly, upper-arm measurements are affected by the patient’s age and sex. Elderly women consistently have larger triceps skinfold thickness, but smaller mid-upper arm muscle area. Similarly, the female triceps skin fold exceeds that of males by up to 83% [67,68]. Hence, it is important to consider these factors when determining upper arm measurements in malnourished patients.

The reliability of upper arm measurement as a nutritional assessment tool has been demonstrated in several studies. In 40 cirrhotic patients, Fiore *et al.* compared skinfold thickness to dual-energy X-ray absorptiometry (DEXA) to evaluate body fat [64]. The authors concluded that skin fold measurements are an accurate method for estimating total body fat and are comparable to previously accepted standardized methods. Similarly, Kamimura *et al.* analyzed skinfold thickness in 30 patients undergoing hemodialysis and concluded that this method was comparable to the reference method DEXA and preferred over bioelectrical impedance analysis (BIA) [66]. Hence, muscle mass and subcutaneous fat measurements are reliable assessment tools for assessing a patient’s nutritional status.

### Hand-grip strength

Malnutrition has also been well correlated with a decrease in muscle strength and overall functional status [69]. Physiologically, malnutrition results in decreased whole body protein concentrations and body cell mass. Specifically, protein synthesis decreases as proteolytic mechanisms are stimulated. Decreased mitochondrial complex activities also contribute to reduced muscle function and impaired free-energy change [70–75]. Overall, these processes result in degeneration of muscle mass. Hence, decrease in muscle strength has been strongly associated with a loss of functional status, and hand-grip strength (HGS) is proposed as an objective surrogate marker for detecting malnutrition [76–78]. Compared with other muscle strength exercises such as hip flexion strength, HGS has been validated as a rapid, cost-effective, and reliable tool for diagnosing patients with malnutrition [79,80]. Furthermore, its high inter-rater reliability as well as its retest reliability are attractive features for regular use of HGS in malnourished patients. Interestingly, HGS has also been reported to be superior to the routine use of the SGA in a cross-sectional study evaluating nutritional status in an outpatient cohort of 50 cirrhotic patients [81].

Several studies have demonstrated the importance of measuring HGS for diagnosing malnutrition in the hospital setting [82–84]. In a study of 287 consecutively admitted patients, Norman *et al.* reported a significant decline in voluntary HGS in malnourished patients compared with their well-nourished counterparts (45.22 kg *vs* 30.82 kg in men) [71]. HGS correlated positively with total body cell mass as well as BMI. Similarly, in 94 patients with Crohn’s disease in clinical remission, HGS also correlated well with body cell mass and was reduced in these patients, despite their having been classified as well-nourished according to the SGA, BMI and serum albumin levels [82]. Futhermore, Vaz *et al.*, in their study of 72 young adult males reported that, HGS was able to differentiate between underweight and chronically energy-deficient patients with similar BMI regardless of total body cell mass [83]. Lastly, in a cross-sectional study of 217 well-nourished and malnourished hospitalized patients, HGS measurements correlated strongly with PG-SGA scores and independently predicted nutritional status and change in nutritional status [84]. HGS measurements were concluded to be independent predictors of overall nutritional status in hospitalized patients. Overall, HGS had a sensitivity and specificity of 86.7% and 70.2%, respectively, for identifying patients with malnutrition [85].

The importance of HGS in malnutrition has also been demonstrated by the recovery of muscle function upon nutritional intervention [86–89]. Paton *et al.* provided nutritional supplementation to 36 patients within 2 weeks of starting anti-tuberculous therapy [86]. Physical strength was measured by maximum voluntary HGS. Compared with non-interventional control subjects, nutritionally supplemented patients had a significant increase in HGS (2.79 +/− 3.11 compared with −0.65 +/− 4.48 kg, *P*= 0.016) at 6 weeks. Similar results were obtained in a study of 124 high-risk acute stroke patients randomized to either individualized nutritional care or routine hospital care [87]. In response to nutritional intervention, there was a significant increase in HGS as well as overall quality of life for those patients.

### Guidelines

The A.S.P.E.N. guidelines for diagnosing malnutrition, which looked at six characteristics, were first proposed in 2009 (Table 2). At least two of the six characteristics are needed for the diagnosis of malnutrition. If two or more characteristics are met, the malnutrition can then be categorized first by severity and further by acuity. For example, weight loss > 2% per week is classified as acute severe malnutrition, while loss of 1–2% per week is considered to be moderate severity. In order to ascertain whether a patient has inadequate energy intake, questions in the history regarding dietary intake are either directed to the patient or the caregiver in cases in which the patient is unable to provide information. The total intake is then compared with the patient’s estimated energy requirements to determine whether he or she is indeed getting sufficient amounts. Intake ≤ 50% for ≥ 5 days is considered severe acute malnutrition, and intake ≤ 75% for ≥ 1 month is severe chronic malnutrition. Weight loss determination requires using the patient’s “dry” weight because fluid overload may hide weight loss.

**Table 2.** A.S.P.E.N guidelines

**A.S.P.E.N. Guidelines**
Insufficient energy intake
Weight loss
Loss of muscle mass
Loss of subcutaneous fat
Local/generalized fluid accumulation
Diminished functional status

A thorough physical examination is necessary to look for specific signs of muscle and fat loss. During severe wasting states, the temples may become hollowed. In addition, ribs may start protruding in the clavicle/pectoralis muscle areas. Muscles in the lower body are more resistant to change, but clinicians should assess the posterior calf and anterior thigh areas by grasping the muscles. Similarly, signs of subcutaneous fat loss include hollows and dark circles in the orbital region, obvious depressions between the ribs and a prominent iliac crest. Examination for the accumulation of fluid should start by assessing a patient’s overall fluid status. This will be especially difficult in patients with diseases such as congestive heart failure, cirrhosis or renal failure. Areas that should not be missed during the examination are the face, abdomen, extremities and scrotum. Pitting edema of 1+, 2+, 3+ and 4+ correlate with 2mm, 4mm, 6mm and 8mm depression, respectively. Diminished functional status is usually assessed with HGS using a dynamometer. It must be remembered that patients with rheumatoid arthritis, stroke and any neuromuscular diseases in addition to those in the ICU may not be able to give valid measurements. If HGS cannot be performed, physicians can use other parameters (eg, activities of daily living) to determine patients’ functional ability.

## Summary

Pros and cons of some frequently used serum markers are summarized in Table 3. Although historically popular, studies are inconsistent for proving the validity of serum markers as determinants of patients’ nutritional status. The major consensus in the literature is that these laboratory markers are not reliable by themselves. They are popular because they offer objective and quantitative results; however, they should only be used as a complement to findings from a thorough physical examination. Furthermore, serum proteins such as albumin are good for detecting inflammatory states rather than malnutrition; the distinction between the two is important for clinicians to understand. Hence, physical examination is a better tool for diagnosing malnutrition. Newer techniques such as BIA are being investigated for their usefulness in determining nutritional status; they also have the advantage of being noninvasive and relatively cost-effective, which allows for simple bedside determination of a patient’s muscle mass. However, it is still uncertain if these techniques are superior to physical examination. What is certain is that malnutrition is an important risk factor for both patient morbidity and increased healthcare costs. With increasing awareness as well as regulations and guidelines set forth by CMS and A.S.P.E.N., respectively, it will no longer be “the skeleton in the hospital closet” [90].

**Table 3.** Pros and cons of serum nutritional markers

**Nutritional marker**	**Pros**	**Cons**
Albumin	Ease of measurement Low cost Reproducibility Excellent predictor of surgical outcomes Consistent response to interventions	Long half-life Decreased levels in infection, burns, fluid overload, hepatic failure, cancer and nephrotic syndrome
Transferrin	Shorter half-life (8–10 days) • Responds more rapidly to changes in protein status	Influenced by several factors including liver disease, fluid status, stress and illness Unreliable in the assessment of mild malnutrition and its response to nutritional intervention Expensive
Prealbumin	Half-life of prealbumin (2–3 days) is much shorter than that of albumin, Easily available Expected to change more rapidly with changes in nutrient intake Unaffected by hydration status	Levels may be increased in the setting of renal dysfunction, corticosteroid therapy Physiological stress, infection and liver dysfunction can decrease prealbumin levels

*Conflict of interest statement*: none declared.
